# Effect of Cu Substitution and Heat Treatment on Phase Formation and Magnetic Properties of Sm_12_Co_88−x_Cu_x_ Melt-Spun Ribbons

**DOI:** 10.3390/ma15134494

**Published:** 2022-06-25

**Authors:** Feilong Dai, Peipei Liu, Lin Luo, Dekang Chen, Qingrong Yao, Jiang Wang

**Affiliations:** 1 Guangxi Key Laboratory of Information Materials, School of Materials Science and Engineering, Guilin University of Electronic Technology, Guilin 541004, China; daifeilong190@163.com (F.D.); lpp656357819@163.com (P.L.); luolin152@163.com (L.L.); chendekang181@163.com (D.C.); qingry96@guet.edu.cn (Q.Y.); 2 Engineering Research Center of Electronic Information Materials and Devices, Ministry of Education, Guilin University of Electronic Technology, Guilin 541004, China

**Keywords:** Sm-Co-Cu, melt-spun ribbons, phase structure, magnetic properties

## Abstract

The phase structure and microstructure of Sm_12_Co_88−x_Cu_x_ (x = 0, 2, 4, 6, 8, 10; at.%) as-cast alloys and melt-spun ribbons prepared via the arc-melting method and melt-spun technology were studied experimentally by X-ray diffraction (XRD) and scanning electron microscope (SEM) with energy dispersive spectroscopy (EDS). The results reveal that the Sm_12_Co_88−x_Cu_x_ (x = 0) as-cast alloy contains Sm_2_Co_17_ and Sm_5_Co_19_ phases, while the Sm_12_Co_88−x_Cu_x_ (x = 2) as-cast alloy is composed of Sm_2_Co_17_, Sm_2_Co_7_ and Sm(Co, Cu)_5_ phases. Sm_2_Co_17_ and Sm(Co, Cu)_5_ phases are detected in Sm_12_Co_88−x_Cu_x_ (x = 4, 6, 8, 10) as-cast alloys. Meanwhile, Sm_12_Co_88−x_Cu_x_ ribbons show a single SmCo_7_ phase, which is still formed in the ribbons annealed at 1023 K for one hour. After annealed at 1123 K for two hours, cooled slowly down to 673 K at 0.5 K/min and then kept for four hours, the ribbons are composed of Sm_2_Co_17_ and Sm(Co, Cu)_5_ phases. The magnetic measurements of Sm_12_Co_88−x_Cu_x_ ribbons were performed by vibrating sample magnetometer (VSM). The results exhibit that the maximum magnetic energy product ((BH)_max_), the coercivity (H_cj_) and the remanence (B_r_) of the Sm_12_Co_88−x_Cu_x_ ribbons increase generally with the increase in Cu substitution. In particular, the magnetic properties of the ribbons annealed at 1123 K and 673 K increase significantly with the increase in Cu substitution, resulting from the increase in the volume fraction of the formed Sm(Co, Cu)_5_ phase after heat treatment.

## 1. Introduction

Sm-Co-based permanent magnets have been used in aerospace, electric vehicle motors, wind turbines, sensors and actuators because of high Curie temperatures, excellent temperature stability and good corrosion resistance [1,2,3]. Extensive investigations were conducted to investigate the magnetic properties of Sm-Co alloys with alloying elements Fe, Cu and Zr, which results in a new class of Sm(Co, Fe, Cu, Zr)_z_ (5 ≤ Z ≤ 8.5) permanent magnets [4,5,6,7,8,9,10,11]. It was reported that the addition of Cu into Sm-Co-based permanent magnets would improve their coercivity (H_cj_) [12,13,14,15,16,17,18]. Tellez-Blanco et al. [14] found that the coercivity of SmCo_5-x_Cu_x_ annealed alloys increases and then decreases with the increase in Cu substitution. Horiuchi et al. [15] reported the (BH)_max_ of Sm(Co_bal_Fe_0.35_Cu_0.06_Zr_0.018_)_7.8_ magnet is 32 MGOe. Subsequently, Horiuchi et al. [16] further improved the H_cj_ and (BH)_max_ of Sm(Co_bal_Fe_0.35_Cu_0.06_Zr_0.018_)_7.8_ magnet by means of process optimization. Wang et al. [17] found that the H_cj_ of Sm(Co_0.665_Fe_0.25_Cu_0.06_Zr_0.025_)_7_ magnet increases significantly from 12 kOe to 21 kOe. Xu et al. [18] investigated the effect of heat treatment on the formation of 1:5H cell wall phase in 2:17 type melt-spun ribbons with high Fe content. It was detected that the Cu content is the key to the formation of the 1:5H cell wall phase, which would improve the magnetic properties of the 2:17 type melt-spun ribbons with high Fe content.

Therefore, in order to further understand the effect of Cu substitution and heat treatment on the magnetic properties of Sm-Co alloys, the phase formation, microstructure and magnetic properties of Sm_12_Co_88−x_Cu_x_ alloys were investigated in this work by X-ray diffraction (XRD), scanning electron microscope (SEM) with energy dispersive spectroscopy (EDS) and vibrating sample magnetometer (VSM).

## 2. Experimental Procedure

Sm_12_Co_88−x_Cu_x_ (x = 0, 2, 4, 6, 8, 10; at.%) alloys were fabricated through arc-melting method using bulk Sm, Co and Cu metals (99.99% purity) as the raw materials. The as-cast alloys were melted four times during the arc-melting process to ensure composition homogeneity. The ribbons were acquired by induction melting as-cast alloys and then spraying the melts through the orifice (orifice diameter approximately 0.8–1.0 mm) onto the copper wheel surface at the wheel speed of 40 m/s. The thickness and the width of the melt-spun ribbons are about 10–15 mm and 2–3 mm, respectively. The ribbons were sealed in quartz tubes filled with high pure argon gas. After heat treatment, the quartz tubes with the ribbons were quenched in ice water. In this work, the first heat-treatment was that the ribbons were annealed at 1023 K for 1 h, while the second heat-treatment was that the ribbons were annealed at 1123 K for 2 h, and then were slowly cooled down to 673 K at 0.5 K/min and kept at 673 K for 4 h.

The phase structure, phase composition and microstructure of as-cast alloys and melt-spun ribbons were analyzed by X-ray powder diffraction (XRD, PLXcel 3D, Cu Kα radiation) and scanning electron microscope with energy dispersive spectroscopy (SEM-EDS, FEI 450G). The lattice parameters and volume fractions of the formed phases in as-cast alloys and the ribbons were determined by the Rietveld refinements with the Fullprof program. The magnetic properties of the ribbons are measured at room temperature via vibrating sample magnetometer (VSM, Lakeshore Model 7400 740H). In this work, the demagnetization correction of the ribbons is neglected because the applied external field is parallel to the plane of the ribbons in the magnetic measurements.

## 3. Results and Discussion

### 3.1. Phase Formation

**Figure 1:**
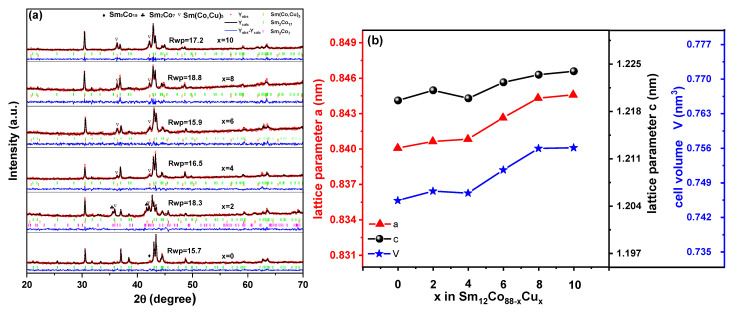
(**a**) Rietveld refinements of XRD patterns of Sm_12_Co_88−x_Cu_x_ (x = 0, 2, 4, 6, 8, 10) as-cast alloys and (**b**) the lattice parameters and cell volume of Sm_2_Co_17_ phase in Sm_12_Co_88−x_Cu_x_ (x = 0, 2, 4, 6, 8, 10) as-cast alloys.

Figure 1a is the Rietveld refinements of XRD powder patterns of Sm_12_Co_88−x_Cu_x_ (x = 0, 2, 4, 6, 8, 10) as-cast alloys. As can be seen in Figure 1a, the red points and solid lines show the experimental and calculated XRD patterns of the as-cast alloys, respectively. The vertical bars indicate Bragg reflection positions. The green lines show the differences between the experimental and computed intensities. Based on the agreement factor (R_wp_), the calculated patterns are consistent with the experimental patterns. On the basis of the Rietveld refinements in Figure 1a, the Sm_12_Co_88−x_Cu_x_ (x = 0) as-cast alloy shows Sm_2_Co_17_ and Sm_5_Co_19_ phases, while the Sm_12_Co_88−x_Cu_x_ (x = 2) as-cast alloy is composed of Sm_2_Co_17_, Sm_2_Co_7_ and Sm(Co, Cu)_5_ phases, and the Sm_12_Co_88−x_Cu_x_ (x = 4, 6, 8, 10) as-cast alloys consist of Sm_2_Co_17_ and Sm(Co, Cu)_5_ phases. Table 1 shows the lattice parameters and the cell volume of the Sm_2_Co_17_ phase in Sm_12_Co_88−x_Cu_x_ as-cast alloys obtained finally by the Rietveld refinements. As can be seen from Figure 1b, except for the Sm_12_Co_88−x_Cux (x = 2) as-cast alloy, the lattice parameters and cell volume of the Sm_2_Co_17_ phase in the Sm_12_Co_88−x_Cu_x_ as-cast alloy increase linearly with the increase in Cu substitution. It means that Cu atoms with the larger radii enter into the structure lattice of the Sm_2_Co_17_ phase for replacing Co atoms in the Sm_12_Co_88−x_Cu_x_ as-cast alloys. However, the lattice parameters and the cell volumes of the Sm_2_Co_17_ phases in the Sm_12_Co_88−x_Cu_x_ (x = 2) as-cast alloy are abnormally larger than those in the samples with more Cu substitution. Guo et al. [19] and Chang et al. [20] showed that Cu atoms tend to occupy the 3g crystal site in RE/(Co, M) structures, and there are much more Sm atoms on the 3g crystal site. Thus, the abnormal increase in the lattice parameters of the Sm_2_Co_17_ phase in the Sm_12_Co_88−x_Cu_x_ (x = 2) as-cast alloy may be caused by the substitution of Sm by Cu at the 3g crystal site. The peak of the Sm(Cu,Co)_5_ phase in the Sm_12_Co_88−x_Cu_x_ (x = 2) as-cast alloy (near 36°) migrates a little compared with other samples. This may be because Co-Co atom pairs replace part of the Sm atoms. Since the atomic radii of the Co-Co atom pairs are larger than those of the Sm atoms, the diffraction peaks of the Sm(Cu,Co)_5_ phase in the Sm_12_Co_88−x_Cu_x_ (x = 2) as-cast alloy shift slightly to lower 2θ values. This phenomenon was introduced by Yuan et al. [21].

**Figure 2:**
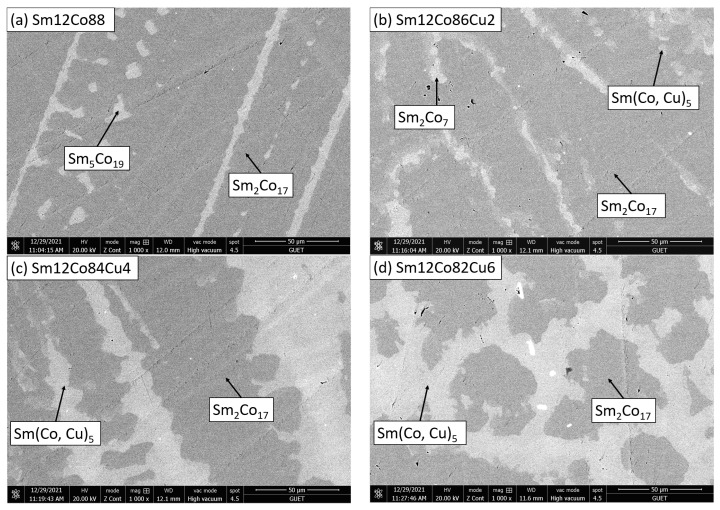

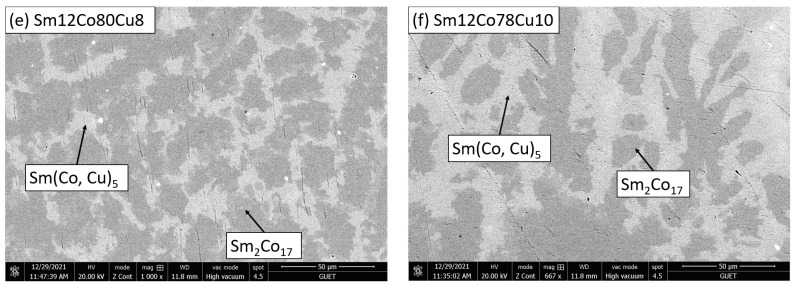
Backscattered electron (BSE) images of Sm_12_Co_88−x_Cu_x_ as-cast alloys. (**a**) x = 0, (**b**) x = 2, (**c**) x = 4, (**d**) x = 6, (**e**) x = 8, (**f**) x = 10.

Figure 2 displays the backscattered electron (BSE) images of Sm_12_Co_88−x_Cu_x_ (x = 0, 2, 4, 6, 8, 10) as-cast alloys. The phase compositions of the formed phases in Sm_12_Co_88−x_Cu_x_ as-cast alloys were measured by EDS as shown in Table 2. In Figure 2a, there are two phases in the microstructure of the Sm_12_Co_88−x_Cu_x_ (x = 0) as-cast alloy. The white phase is the Sm_5_Co_19_ phase, and the gray phase is the Sm_2_Co_17_ phase based on the EDS measurements in Table 2. It was stated clearly that the SEM-EDS results of Sm_12_Co_88−x_Cu_x_ as-cast alloys are consistent with the XRD results, except for the Sm_5_Co_19_ phase in the Sm_12_Co_88−x_Cu_x_ (x = 0) as-cast alloy, which was not detected by the XRD analysis due to low volume fraction.

**Figure 3:**
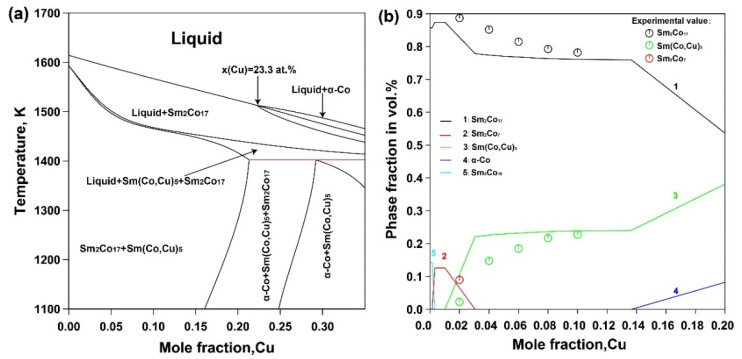
(**a**) Calculated vertical section of 12 at.% Sm in the Sm-Co-Cu ternary system and (**b**) calculated phase fractions as a function of Cu content in Sm_12_Co_88−x_Cu_x_ as-cast alloys at 673 K.

Figure 3 shows the calculated vertical section of 12 at.% Sm and volume fractions of the formed phases as a function of Cu concentration in the Sm_12_Co_88−x_Cu_x_ as-cast alloys at 673 K, which were calculated with the thermo-Calc^®^ software package using thermodynamic parameters of the Sm-Co-Cu ternary system developed by Dai et al. [22]. As can be seen in Figure 3a, during the solidification process, the Sm_2_Co_17_ phase is precipitated firstly from the liquid phase of the Sm_12_Co_88−x_Cu_x_ alloys with low Cu content (<24 at.%) and then the Sm(Co, Cu)_5_ phase is formed. In contrast, the α-Co phase is precipitated firstly from the liquid phase of the Sm_12_Co_88−x_Cu_x_ alloys with high Cu content (>24 at.%) and then the Sm_2_Co_17_ and Sm(Co, Cu)_5_ phases are formed. In Figure 3b, it was found that the calculated volume fractions of the formed phases as a function of Cu substitution are consistent with the experimental results determined by the Rietveld refinements as shown in Table 1. The Sm_5_Co_19_ phase formed in the Sm_12_Co_88−x_Cu_x_ as-cast alloys would change to the Sm_2_Co_7_ phase with the increase in Cu substitution, while the stable Sm(Co, Cu)_5_ phase is formed and the Sm_2_Co_7_ phase disappears. Meanwhile, the volume fraction of the Sm_2_Co_17_ phase decreases, while that of the Sm(Co, Cu)_5_ phase decreases. The formation of Sm_5_Co_19_ and Sm_2_Co_7_ phases in the Sm_12_Co_88−x_Cu_x_ as-cast alloys is inhibited with the increase in Cu substitution, which is effective for the formation of the Sm(Co, Cu)_5_ phase in as-cast alloys.

**Figure 4:**
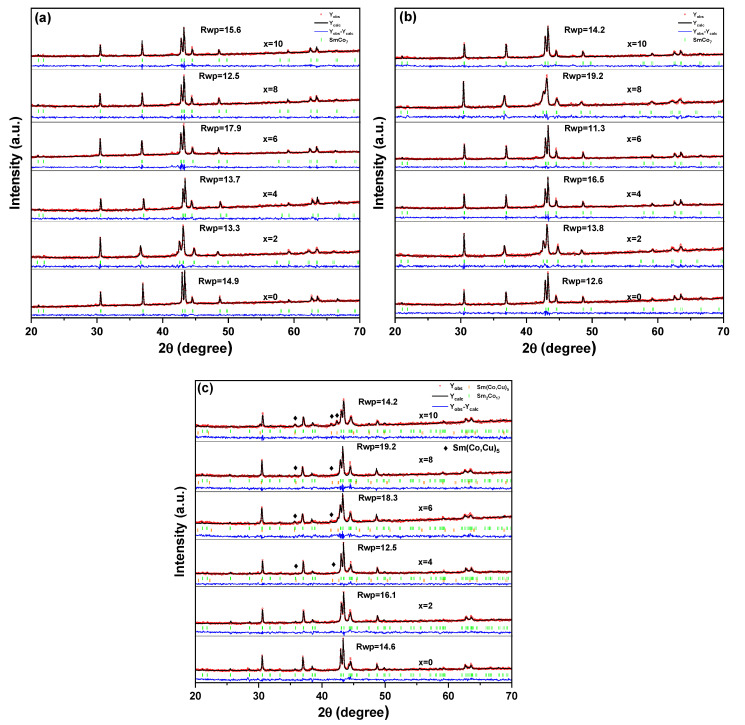
Rietveld refinements of XRD patterns of Sm_12_Co_88−x_Cu_x_ (x = 0, 2, 4, 6, 8, 10) melt-spun ribbons. (**a**) Melt-spun ribbons, (**b**) Melt-spun ribbons annealed at 1023 K, (**c**) Melt-spun ribbons annealed at 1123 K and 673 K.

Figure 4 is the Rietveld refinements of the XRD powder patterns of Sm_12_Co_88−x_Cu_x_ (x = 0, 2, 4, 6, 8, 10) melt-spun ribbons. The lattice parameters, cell volumes and volume fractions of the formed phases in the Sm_12_Co_88−x_Cu_x_ melt-spun ribbons obtained by the Rietveld refinements are given in Table 1. In Figure 4a, the ribbons show a single SmCo_7_ phase, and the ribbons annealed at 1023 K for 1 h are still a single SmCo_7_ phase in Figure 4b. For melt-spun ribbons and the melt-spun annealed at 1023 K, it can be seen from Table 1 that the lattice parameters and cell volumes of the ribbons with x = 2, 4, 6, 8, 10 are greater than the ribbon with x = 0. It means that Cu atoms with larger radius enter into the structure lattice of the SmCo_7_ phase for replacing Co atoms in the Sm_12_Co_88−x_Cu_x_ melt-spun ribbons and the Sm_12_Co_88−x_Cu_x_ melt-spun ribbons annealed at 1023 K. Figure 4c exhibits that the ribbons annealed at 1123 K and 673 K show the formation of the Sm_2_Co_17_ and Sm(Co, Cu)_5_ phases, and the disappearance of the SmCo_7_ phase. It was found that Sm_12_Co_88−x_Cu_x_ (x = 0, 2) melt-spun ribbons are a single Sm_2_Co_17_ phase, while Sm_12_Co_88−x_Cu_x_ (4, 6, 8, 10) melt-spun ribbons are composed of Sm_2_Co_17_ and Sm(Co, Cu)_5_ phases. Furthermore, the volume fraction of the Sm(Co, Cu)_5_ phase in the ribbons annealed at 1123 K and 673 K increases with the increase in Cu substitution, while that of the Sm_2_Co_17_ phase decreases. Furthermore, the lattice parameters and cell volumes of Sm_2_Co_17_ phases formed in Sm_12_Co_88−x_Cu_x_ ribbons annealed at 1123 K and 673 K increase generally with the increase in Cu substitution, indicating that Cu atoms with larger radii enter the structure lattice of the Sm_2_Co_17_ phase for replacing Co atoms.

### 3.2. Magnetic Properties

**Figure 5:**
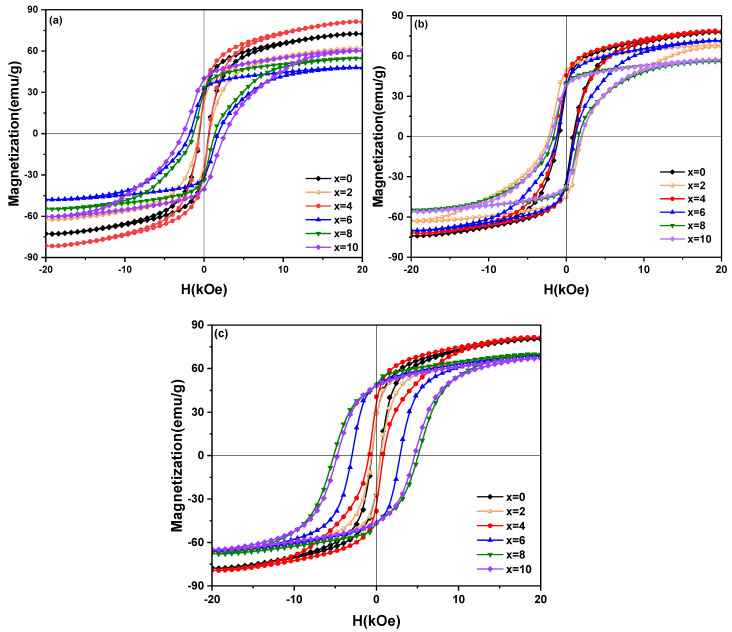
Hysteresis loops (M-H curves) of Sm_12_Co_88−x_Cu_x_ (x = 0, 2, 4, 6, 8, 10) melt-spun ribbons with different heat treatments. (**a**) Melt-spun ribbons, (**b**) Melt-spun ribbons annealed at 1023 K, (**c**) Melt-spun ribbons annealed at 1123 K and 673 K.

**Figure 6:**
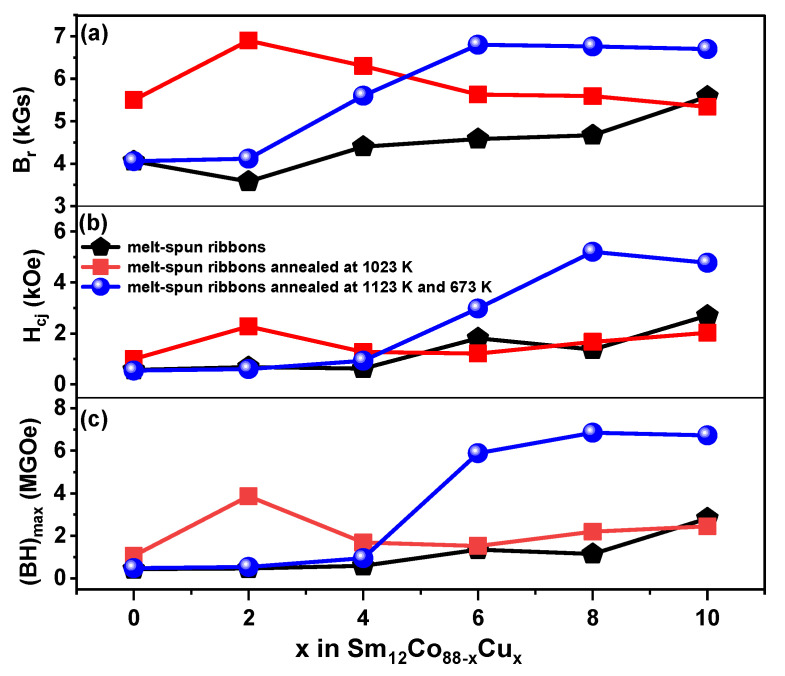
Magnetic properties as a function of Cu content in Sm_12_Co_88−x_Cu_x_ (x = 0, 2, 4, 6, 8, 10) melt-spun ribbons. (**a**) Melt-spun ribbons, (**b**) Melt-spun ribbons annealed at 1023 K, (**c**) Melt-spun ribbons annealed at 1123 K and 673 K.

Figure 5 shows the hysteresis loops of the Sm_12_Co_88−x_Cu_x_ (x = 0, 2, 4, 6, 8, 10) melt-spun ribbons. Based on the hysteresis loops (M-H curves) as shown in Figure 5, the remanence (B_r_) and coercivity (H_cj_) of the ribbons were obtained, and the maximal magnetic energy product ((BH)_max_) was determined as the area of the biggest rectangle that is inscribed in the second quadrant of B-H curves transformed from M-H curves. The magnetic properties (B_r_, H_cj_ and (BH)_max_) of the Sm_12_Co_88−x_Cu_x_ ribbons determined in this work are summarized in Table 3 as shown in Figure 6. Figure 6a shows the B_r_ of the ribbons; the ribbons annealed at 1123 K and 673 K increase normally with the increase in Cu substitution, while that of the ribbons annealed at 1023 K increases and then decreases. In Figure 6b,c, the H_cj_ and (BH)_max_ of the ribbons show similar tendencies with the increase in Cu substitution. The reason for it could be that the formation of the SmCo_5_ phase in Sm-Co alloys can enhance the pinning effect of domain walls, and improve the magnetic properties of the alloys, which was reported by Xu et al. [18], Cao et al. [23] and Xia et al. [24]. The XRD results in Figure 4c show that the Sm(Co, Cu)_5_ phase is formed in the Sm_12_Co_88−x_Cu_x_ (x = 4, 6, 8, 10) ribbons annealed at 1123 K and 673 K. Furthermore, the volume fraction of the Sm(Co, Cu)_5_ phase in the ribbons annealed at 1123 K and 673 K increases with the increase in Cu substitution. The increase in the volume fraction of the Sm(Co, Cu)_5_ phase is an important factor for the significant increase in the H_cj_ and (BH)_max_ of the ribbons annealed at 1123 K and 673 K.

Compared with the magnetic properties of the ribbons under different heat treatments in Figure 6, it was found that the B_r_, H_cj_ and (BH)_max_ of the Sm_12_Co_88−x_Cu_x_ (x = 0, 2) ribbons annealed at 1023 K are better than those of the unannealed ribbons. This could be due to the fact that the alloy composition and microstructure are much more uniform after heat treatment. However, after annealing at 1023 K, the magnetic properties of the Sm_12_Co_88−x_Cu_x_ (x = 6, 8, 10) decreased. This could be due to the excessively large grain size and microstructure deterioration resulting in the reduction in remanence and coercivity during excessive heat treatment at a high temperature, which is similar to the results of Feng et al. [25]. In particular, after being annealed at 1123 K and 673 K, the B_r_, H_cj_ and (BH)_max_ of Sm_12_Co_88−x_Cu_x_ (x = 6, 8, 10) ribbons with high Cu content are much better, and increase generally with the increase in Cu substitution. The Sm_12_Co_88−x_Cu_x_ (x = 2) ribbon annealed at 1023 K presents optimal magnetic properties (B_r_ = 6.91 kGs, H_cj_ = 2.28 kOe, (BH)_max_ = 3.86 MGOe), while the best magnetic properties (B_r_ = 6.76 kGs, H_cj_ = 5.20 kOe and (BH)_max_ = 6.85 MGOe) of the Sm_12_Co_88−x_Cu_x_ (x = 8) ribbons annealed at 1123 K and 673 K were obtained in this work. Therefore, the magnetic properties of Sm_12_Co_88−x_Cu_x_ ribbons determined in this work as a function of Cu substitution indicate that the heat treatment is a promising and effective way to enhance the magnetic properties of Sm-Co-Cu melt-spun ribbons.

**Figure 7:**
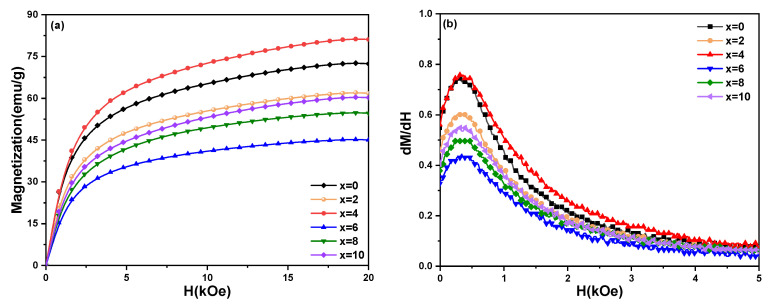

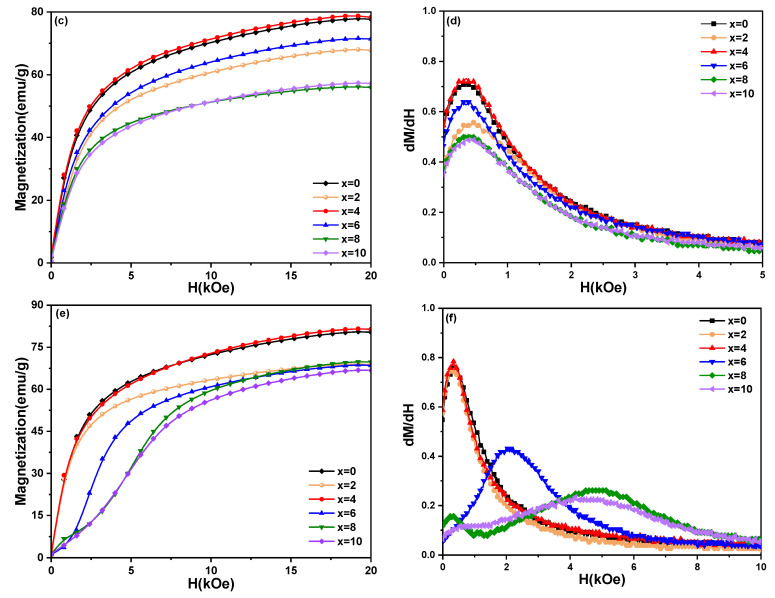
(**a**,**c**,**e**) The initial magnetization curves and (**b**,**d**,**f**) the first derivatives of the initial magnetization curve of Sm_12_Co_88−x_Cu_x_ (x = 0, 2, 4, 6, 8, 10) melt-spun ribbons with different heat treatments. (**a**,**b**) Melt-spun ribbons, (**c**,**d**) Melt-spun ribbons annealed at 1023 K, (**e**,**f**) Melt-spun ribbons annealed at 1123 K and 673 K.

In order to understand further the magnetization behavior of the ribbons, the initial magnetization curves and the first derivatives of the initial magnetization curve of Sm_12_Co_88−x_Cu_x_ ribbons was measured at room temperature as shown in Figure 7. In Figure 7a, the initial magnetization of Sm_12_Co_88−x_Cu_x_ ribbons increases rapidly with the increase in the external magnetic field, implying that the magnetization process of the ribbons is controlled by nucleation. It can be seen from Figure 7b that only one magnetization inversion exists in all the ribbons. Combined with the results of Figure 4a, the ribbons consist of SmCo_7_ single phase, indicating that the magnetization process of Sm_12_Co_88−x_Cu_x_ ribbons is controlled by a pure nucleation mechanism. In Figure 7c,d, the magnetization process of the Sm_12_Co_88−x_Cu_x_ ribbons annealed at 1023 K is still controlled through pure nucleation. Figure 7e exhibits that the magnetization process of the Sm_12_Co_88−x_Cu_x_ (x = 0, 2, 4) ribbons annealed at 1123 K and 673 K increases rapidly with the increase in the external magnetic field. Figure 7f shows that only one magnetization inversion exists in Sm_12_Co_88−x_Cu_x_ (x = 0, 2, 4) ribbons annealed at 1123 K and 673 K, indicating that the magnetization process of the ribbons with low Cu content is controlled by pure nucleation. Nevertheless, there are two types of collective magnetization reversal in the initial magnetization curves of Sm_12_Co_88−x_Cu_x_ (x = 6, 8, 10) ribbons annealed at 1123 K and 673 K in Figure 7e, indicating that the initial magnetization process of these ribbons with high Cu substitution is controlled by both pinning mechanism and nucleation. As can be seen in Figure 7f, when the external magnetic field is low, the local nucleation in Sm_12_Co_88−x_Cu_x_ (x = 6, 8, 10) ribbons may be caused by direct contact of the Sm_2_Co_17_ phase. With the increase in the Sm(Co, Cu)_5_ phase, the domain wall pinning provided by the Sm(Co, Cu)_5_ phase ultimately determines the magnetization inversion of the Sm_12_Co_88−x_Cu_x_ (x = 6, 8, 10) ribbons. It means that the coercivity mechanism of the ribbons annealed at 1123 K and 673 K changes from the nucleation mechanism to the pinning mechanism with the increase in Cu substitution.

## 4. Conclusions

The effects of Cu substitution and heat treatment on the phase formation and magnetic properties of Sm_12_Co_88−x_Cu_x_ melt-spun ribbons were investigated in this work using XRD, SEM-EDS and VSM. The following conclusions could be drawn:

(1) The XRD and SEM-EDS results indicate that the Sm_12_Co_88−x_Cu_x_ (x = 0) as-cast alloy contains Sm_2_Co_17_ and Sm_5_Co_19_ phases, and the Sm_12_Co_88−x_Cu_x_ (x = 2) as-cast alloy is composed of Sm_2_Co_17_, Sm_2_Co_7_ and Sm(Co, Cu)_5_ phases. Both the Sm_2_Co_17_ and Sm(Co, Cu)_5_ phases are detected in the Sm_12_Co_88−x_Cu_x_ (x = 4, 6, 8, 10) as-cast alloys. Meanwhile, Sm_12_Co_88−x_Cu_x_ ribbons show a single SmCo_7_ phase, which is still formed in the ribbons annealed at 1023 K. After being annealed at 1123 K and 673 K, Sm_12_Co_88−x_Cu_x_ (x = 0, 2) ribbons consist of a Sm_2_Co_17_ single phase, while Sm_12_Co_88−x_Cu_x_ (x = 4, 6, 8, 10) ribbons contain Sm_2_Co_17_ and Sm(Co, Cu)_5_ phases.
(2) Magnetic measurements show that the magnetic properties of Sm_12_Co_88−x_Cu_x_ ribbons (x = 4, 6, 8, 10) with high Cu substitution annealed at 1123 K and 673 K are improved significantly, and the coercivity mechanism of these ribbons is controlled by both a pinning mechanism and a nucleation mechanism. The volume fraction of the Sm(Co, Cu)_5_ phase in the ribbons increases after heat treatment, which is an important factor for the enhancement of the coercivity and maximal magnetic energy product. The best magnetic properties with B_r_ = 6.76 kGs, H_cj_ = 5.20 kOe and (BH)_max_ = 6.85 MGOe were achieved in Sm_12_Co_88−x_Cu_x_ (x = 8) ribbons annealed at 1123 K and 673 K.

## Figures and Tables

**Table 1:** Lattice parameters, cell volume and volume fraction of the formed phases in Sm_12_Co_88−x_Cu_x_ alloys by Rietveld refinements.

Sm_12_Co_88−x_Cu_x_ Alloys	x	Lattice Parameters						Volume Fractions			
		Sm_2_Co_17_			SmCo_7_			Sm_2_Co_17_	Sm(Co, Cu)_5_	Sm_2_Co_7_	SmCo_7_
		a (Å)	c (Å)	Volume (Å^3^)	a (Å)	c (Å)	Volume (Å^3^) (Å^3^)	(%)	(%)	(%)	(%)
As cast alloys	0	8.400 (8)	12.196 (2)	745.417 (2)	—	—	—	100	—	—	—
	2	8.406 (4)	12.211 (2)	747.321 (6)	—	—	—	88.7	2.3	9.0	—
	4	8.408 (3)	12.199 (4)	746.931 (9)	—	—	—	85.2	14.8	—	—
	6	8.426 (5)	12.223 (0)	751.626 (2)	—	—	—	81.5	18.5	—	—
	8	8.443 (1)	12.234 (2)	755.958 (6)	—	—	—	79.3	21.7	—	—
	10	8.445 (9)	12.239 (3)	756.100 (0)	—	—	—	78.2	22.8	—	—
Melt-spun ribbons	0	—	—	—	4.857 (8)	4.069 (9)	83.173 (8)	—	—	—	100
	2	—	—	—	4.905 (6)	4.047 (6)	84.355 (0)	—	—	—	100
	4	—	—	—	4.847 (7)	4.080 (6)	83.048 (5)	—	—	—	100
	6	—	—	—	4.880 (1)	4.067 (6)	83.895 (4)	—	—	—	100
	8	—	—	—	4.871 (7)	4.076 (5)	83.789 (3)	—	—	—	100
	10	—	—	—	4.872 (9)	4.070 (7)	83.711 (4)	—	—	—	100
Melt-spun ribbons annealed at 1023 K	0	—	—	—	4.867 (2)	4.067 (6)	83.450 (2)	—	—	—	100
	2	—	—	—	4.905 (4)	4.047 (9)	84.353 (3)	—	—	—	100
	4	—	—	—	4.870 (0)	4.068 (5)	83.565 (4)	—	—	—	100
	6	—	—	—	4.868 (7)	4.068 (3)	83.515 (7)	—	—	—	100
	8	—	—	—	4.912 (8)	4.066 (2)	84.991 (1)	—	—	—	100
	10	—	—	—	4.873 (6)	4.070 (5)	83.729 (6)	—	—	—	100
Melt-spun ribbons annealed at 1123 K and 673 K	0	8.408 (2)	12.200 (7)	746.992 (6)	—	—	—	100	—	—	—
	2	8.416 (4)	12.208 (3)	748.920 (2)	—	—	—	100	—	—	—
	4	8.401 (6)	12.217 (6)	746.866 (6)	—	—	—	95.1	4.9	—	—
	6	8.410 (2)	12.210 (4)	747.948 (4)	—	—	—	93.4	6.6	—	—
	8	8.428 (4)	12.222 (8)	751.961 (7)	—	—	—	88.6	11.4	—	—
	10	8.435 (4)	12.223 (9)	752.855 (4)	—	—	—	84.2	15.8	—	—

**Table 2:** Phase formation and phase composition of Sm_12_Co_88−x_Cu_x_ as-cast alloys determined by EDS and XRD.

Sm_12_Co_88−x_Cu_x_ As-Cast Alloys (at.%)	Composition Measured by EDS (at.%)				Phase Identified by XRD
	Sm	Co	Cu	Phase	
x = 0	20.13	78.87	0	Sm_5_Co_19_	Sm_5_Co_19_
	11.23	88.77	0	Sm_2_Co_17_	Sm_2_Co_17_
x = 2	21.58	77.19	1.23	Sm_2_Co_7_	Sm_2_Co_7_
	14.16	84.19	1.65	Sm(Co, Cu)_5_	Sm(Co, Cu)_5_
	10.69	88.18	1.12	Sm_2_Co_17_	Sm_2_Co_17_
x = 4	15.78	80.40	3.82	Sm(Co, Cu)_5_	Sm(Co, Cu)_5_
	11.62	85.65	2.73	Sm_2_Co_17_	Sm_2_Co_17_
x = 6	15.78	79.27	4.98	Sm(Co, Cu)_5_	Sm(Co, Cu)_5_
	11.05	83.63	5.32	Sm_2_Co_17_	Sm_2_Co_17_
x = 8	16.23	76.65	7.12	Sm(Co, Cu)_5_	Sm(Co, Cu)_5_
	11.39	82.34	6.27	Sm_2_Co_17_	Sm_2_Co_17_
x = 10	16.32	75.22	8.46	Sm(Co, Cu)_5_	Sm(Co, Cu)_5_
	11.22	81.65	7.13	Sm_2_Co_17_	Sm_2_Co_17_

**Table 3:** Magnetic properties of Sm_12_Co_88−x_Cu_x_ melt-spun ribbons.

Sm_12_Co_88−x_Cu_x_ Ribbons	x	B_r_ (kGs)	H_cj_ (kOe)	(BH)_max_ (MGOe)
Melt-spun ribbons	0	4.06	0.57	0.43
	2	3.58	0.68	0.46
	4	4.41	0.62	0.58
	6	4.58	1.81	1.34
	8	4.67	1.37	1.14
	10	5.58	2.70	2.81
Melt-spun ribbons annealed at 1023 K	0	5.50	0.99	1.05
	2	6.91	2.28	3.86
	4	6.33	1.27	1.68
	6	5.63	1.21	1.52
	8	5.59	1.67	2.19
	10	5.34	2.03	2.44
Melt-spun ribbons annealed at 1123 K and 673 K	0	4.06	0.54	0.47
	2	4.12	0.60	0.53
	4	5.61	0.93	0.94
	6	6.82	2.98	5.88
	8	6.76	5.20	6.85
	10	6.70	4.77	6.72

## Data Availability

Data sharing is not applicable to this article.
